# Validation of an educative manual for patients with head and neck cancer submitted to radiation therapy**^1^**

**DOI:** 10.1590/1518-8345.0949.2706

**Published:** 2016-06-14

**Authors:** Flávia Oliveira de Almeida Marques da Cruz, Elaine Barros Ferreira, Christiane Inocêncio Vasques, Luciana Regina Ferreira da Mata, Paula Elaine Diniz dos Reis

**Affiliations:** 2 Master's student, Departamento de Enfermagem, Universidade de Brasília, Brasília, SP, Brazil.; 3 PhD, Adjunct Professor, Faculdade de Ciências da Saúde, Universidade de Brasília, Brasília, DF, Brazil; 4 Adjunct Professor, Universidade Federal São João Del-Rei, Divinópolis, MG, Brazil.

**Keywords:** Oncology Nursing, Health Education, Nursing Care, Validation Studies, Educational Technology

## Abstract

**Objective:**

develop the content and face validation of an educative manual for patients with head and neck cancer submitted to radiation therapy.

**Method:**

descriptive methodological research. The Theory of Psychometrics was used for the validation process, developed by 15 experts in the theme area of the educative manual and by two language and publicity professionals. A minimum agreement level of 80% was considered to guarantee the validity of the material.

**Results:**

the items addressed in the assessment tool of the educative manual were divided in three blocks: objectives, structure and format, and relevance. Only one item, related to the sociocultural level of the target public, obtained an agreement rate <80%, and was reformulated based on the participants' suggestions. All other items were considered appropriate and/or complete appropriate in the three blocks proposed: objectives - 92.38%, structure and form - 89.74%, and relevance - 94.44%.

**Conclusion:**

the face and content validation of the educative manual proposed were attended to. This can contribute to the understanding of the therapeutic process the head and neck cancer patient is submitted to during the radiation therapy, besides supporting clinical practice through the nursing consultation.

## Introduction

The term head and neck cancer represents the malign tumors of the upper aerodigestive tract, including the oral cavity, pharynx and larynx^1^. The location of the disease imposes intense physical, social and psychological suffering, in view of the changes caused in the individual's basic functions, such as eating, breathing and speech^2^ ^-^ ^3^.

Radiation therapy in the head and neck region is related to some adverse effects, such as mucositis, xerotomy, changes in the sense of taste, dental caries and radiodermatitis, which can trigger a negative impact on these individuals' quality of life^1^. It is essential that nurses not only maintain their role in the maintenance of the treatment and its adverse effects, but also act as information disseminators about the disease and its treatment, offering relief measures and helping the patients to cope with the disease^4^. Therefore, the challenge of nursing care for this population relates to the different physical and psychosocial demands, which need to be attended to through different forms of communication and counseling, besides specialized theoretical and practical knowledge involving care^5^.

The elaboration of new care strategies configures nursing as a science under construction. The constant advance in the nursing work process stimulates the construction of technologies aimed at systemizing and making nursing actions more effective^6^. Hence, the development of health technologies is pertinent to provide the orientations needed to control the adverse effects of the radiation therapy for the head and neck cancer patients. Educative tools play an important role as a support strategy for health education activities, as they help people to understand the information that is transmitted, besides serving as a readily available resource for patients and families to use at home^7^. The print material can facilitate the patient's learning and knowledge dissemination, significantly contributing to nursing work by highlighting and supporting nursing care, besides developing standardized nursing team orientations^8^ and favoring self-care.

To guarantee the achievement of these objectives, however, this material needs to be tested to discover its effectiveness^9^. Since 1990's, health technologies have been elaborated that were acknowledged and used after a validation process^10^. Submitting a tool to the validation process is fundamental to verify the quality of the information and thus establish the use of the material at the health service, supporting team care and the nurses' educative role^11^.

In view of the importance of guaranteeing the validity of the material before its use, the objective in this research was to develop the face and content validation of the educative material focused on head and neck cancer patients submitted to radiation therapy.

## Method

In this descriptive and methodological research, the development, assessment and improvement of a methodological strategy were emphasized^12^. The Theory of Psychometrics was used, which is based on three hubs: theoretical, experimental and analytic^13^. The theoretical hub, focused on the preliminary background and validation of the construct, granting it theoretical quality, is very relevant for the development of research focused on the construction and validation of tools. Therefore, only the procedures in that hub were addressed in this study, which was adapted because the proposed tool is an educative manual and not a psychometric scale, as that theory proposed^13^. 

The theoretical procedures to construct the educative manual started with a bibliographic survey about cancer, radiation therapy, its adverse effects and the care needed to prevent it, besides other relevant issues related to the theme. These topics were taken from different sources, including scientific articles, technical books, manuals from the National Cancer Institute José Alencar Gomes da Silva (INCA), which were added to the researchers' experience at the radiation therapy outpatient clinic.

The manual was called Orientation Manual: radiation therapy in head and neck, and was focused on the patients attended at the High-Complexity Oncology Radiotherapy Outpatient Clinic of the Brasília University Hospital (CACON/HUB), Brasília, DF, Brazil, which offers multidisciplinary outpatient care to individuals diagnosed with malign tumor. The size of the material is 190x280mm and consists of 35 pages, including pretextual (front and back cover, catalographic card, summary and presentation), textual (chapters about cancer, radiation therapy, treatment steps, collateral effects of the radiation therapy and how to prevent them, tracheotomy and nasoenteral tube) and post-textual components (final information and bibliographic references).

The content validation of a tool is judgment based, aiming to test the content addressed in the educative manual and to verify whether the material is appropriate to the detailed concept of the content of interest. The face validation is aimed at verifying whether the manual is understandable to the members of the target population, if it is clear, easy to read and understand. Therefore, experts in the thematic area of the educative material should be capable of assessing it, resulting in its validation^13^.

To choose the study participants, non-probabilistic intentional sampling was used^14^, with a minimum number of six individuals^13^. The subjects were selected based on the contact list of the research group the researchers in this study are members of, through the analysis of the Lattes curriculum of individuals working in the thematic area of the educative manual. The criteria used to select the experts were established, considering the degree, specialization, scientific production, knowledge and length of experience in the theme under discussion, adapted from the expert scoring system adopted in Fehring's model, in which only experts who reached a minimum score of five points were considered apt for inclusion in the expert group for the content and face validation^15^. To improve the face validation, in addition, professionals were selected who work in Publicity and Languages, who assessed the format and linguistic and didactical aspects of the manual, and therefore did not need to comply with the criteria regarding expertise on the theme. 

The participants were formally invited through an invitation letter forwarded by e-mail and, after acceptance, the materials for assessment were also sent by e-mail, including the Informed Consent Form (ICF), the assessment tool and the educative manual. 

The data were collected between October 2014 and April 2015. The sample consisted of 15 experts: nine nurses, three physicians, one dental surgeon, one medical physician and one dosimetrist. Besides these subjects, one grammatical reviewer with a teaching diploma and one advertising agent were also invited to participate in the face validation. The data were collected through an assessment tool, which permitted assessing the content and appearance of the material, adapted from an existing questionnaire^16^ with the author's authorization. The necessary medications were made, considering the theme addressed in the educative manual. The tool was constructed in the form of a Likert scale with five levels of understanding: inappropriate (I), partially inappropriate (PA), I'm not sure (N), appropriate (A) and totally appropriate (TA). The options A and TA were groups to represent the appropriateness of the item, while the options N and PA represented undecided and I represents an invalid item.

To determine the relevance of each item addressed in the assessment tool, a minimum interrater agreement level of 80% was considered^13^. The group including the options A and TA should obtain at least 80% of the answers to guarantee the validation. The item that obtained an agreement level inferior to 80% was reformulated based on the experts' suggestions, in confrontation with the literature and with clinical evidence. The data were types, processed and analyzed through descriptive statistics.

The research project was submitted to the Research Ethics Committee at the School of Health Sciences of the University of Brasília (CEP/FS-UnB), and approved under Opinion 493.456, CAAE: 24592213.1.0000.0030. 

## Results

The sample consisted of 17 subjects (nine nurses, three physicians, one dental surgeon, one medical physician, one dosimetrist, one grammatical reviewer with a teaching diploma in languages and one advertising agent), including 12 women and five men. Ages ranged between 25 and 53 years (mean ()=34.82 years ±7.53), the time since graduation varied between two and 21 years (=11.41±7.68) and the length of experience in the theme area of the educative manual between one and 18 years (=9.35±5.02). Concerning the health professionals (n=15) who were considered experts, after analyzing the Lattes curriculum, according to the criteria of the expert scoring system used in this study, three held a Ph.D., nine a Master's degree and 13 a specialization degree, reminding that a single person could have more than one degree. It should be highlighted that the score varied between 6 and 15 points (=9.73±2.46). The dental surgeon obtained the best score (15 points).

What the validation process of the educative manual is concerned, the experts' opinions (n=15) were analyzed quantitatively, through the answers given to the items of the assessment tool, focusing on three blocks: objectives, structure and form, and relevance. At the end of each block, the experts could justify their answers and/or make suggestions for the educative manual. 

Table 1 presents the experts' answers and the Agreement Rate (AR) of each item in the first assessment block, which consisted of seven items through which the experts' opinion on the objective and goal of the educative manual was verified. All items reached an 80% AR, ranging from 80 to 100%. The mean AR for the block was 92.38%. 

**Table 1:** Experts' assessment of objectives of the educative manual. Brasília, DF, Brazil, 2015

Assessment items	n=15				%^¶^	
	I*	PA^†^	N^‡^	A^§^	TA^||^	
A - O manual é coerente com as necessidades dos pacientes com câncer de cabeça e pescoço, submetidos à radioterapia	0	0	0	3	12	100.00
B - É coerente do ponto de vista do processo de tratamento (etapas da radioterapia)	0	1	0	2	12	93.33
C - É coerente do ponto de vista do processo de educação em saúde (fornece informações e orientações importantes e necessárias)	0	0	0	6	9	100.00
D - É efetivo para a manutenção do autocuidado em domicílio pelo paciente	0	0	2	2	11	86.66
E - É capaz de promover mudanças de comportamento e attitude	0	0	2	6	7	86.66
F - Pode circular no meio científico da área da oncologia e radioterapia	0	0	0	4	11	100.00
G - Atende aos objetivos do CACON/HUB e de outras instituições que trabalham com câncer e radioterapia, podendo o seu uso ser estendido para outros serviços de saúde	0	1	2	2	10	80.00
Total	0	2	6	25	72	92.38

*Inappropriate

†Partially appropriate

‡I am not sure

§Appropriate

||Totally appropriate

¶Agreement rate calculated by adding up the number of appropriate and totally appropriate expert judgments: TA+A x 100/total answers

In Table 2, the experts' answers and the AR for each item in the second block are displayed, which consisted of 13 items, used to verify the experts' opinion on the structure and format of the material. Item B did not reach the minimum AR established, reaching 73.33%. All other items in the block reached the AR of 80%, ranging from 80 to 100%. The mean AR in the block corresponded to 89.74%.

**Table 2:** Experts' assessment on the structure and format of the educative manual. Brasília, DF, Brazil, 2015

Assessment items	n=15				%^¶^	
	I*	PA^†^	N^‡^	A^§^	TA^||^	
A - O manual é apropriado para pacientes com câncer de cabeça e pescoço, submetidos à radioterapia (público-alvo)	0	0	0	5	10	100.00
B - Está apropriado ao nível sociocultural do público-alvo	0	1	3	7	4	73.33
C - É capaz de atingir diferentes camadas socioculturais	0	0	1	7	7	93.33
D - As informações estão apresentadas de maneira clara e objetiva	0	0	1	3	11	93.33
E - As informações apresentadas estão cientificamente corretas	0	1	1	1	12	86.66
F - Há sequência lógica no conteúdo abordado	0	0	1	1	13	93.33
G - As informações estão bem estruturadas em concordância e ortografia	0	2	1	4	8	80.00
H - O estilo da redação corresponde ao nível sociocultural do público-alvo	0	0	3	8	4	80.00
I - O estilo de redação é capaz de atingir diferentes camadas socioculturais	0	0	2	7	6	86.66
J - Informações da capa, contracapa, sumário e apresentação estão coerentes	0	0	1	2	12	93.33
L - O tamanho do título e dos tópicos está adequado	0	0	0	4	11	100.00
M - As ilustrações estão adequadas e em quantidade suficiente	0	0	1	3	11	93.33
N - A quantidade de páginas está adequada	0	0	1	2	12	93.33
Total	0	4	16	54	121	89.74

*Inappropriate

†Partially appropriate

‡I am not sure

§Appropriate

||Totally appropriate

¶Agreement rate calculated by adding up the number of appropriate and totally appropriate expert judgments: TA+A x 100/total answers

From the perspective of Social Communication, the graphical planning of the manual was considered an aligned and practical esthetic unit. The message transmitted was considered concise and clear, which are essential characteristics for good communication. What the linguistic and didactical aspects are concerned, it was considered that the organization and use of the grammatical standards of Portuguese language in the educative manual were of good quality.

In Table 3, the experts' answers and the AR for each item in the third assessment block are displayed, which consisted of six items, assessing the characteristics that determine the degree of significance of the educative material. All items reached an 80% AR, ranging from 86.66 to 100%. The mean AR for the block was 94.44%.

**Table 3:** Experts' assessment on the relevance of the educative manual. Brasília, DF, Brazil, 2015

Assessment items	n=15				%^¶^	
	I*	PA^†^	N^‡^	A^§^	TA^||^	
A - Os temas abordados retratam aspectos essenciais ao autocuidado que devem ser reforçados ao público-alvo	0	0	0	2	13	100.00
B - O manual permite a transferência e generalizações do aprendizado em diferentes contextos (hospitalar e domiciliar)	0	0	0	2	13	100.00
C - O manual é efetivo quando propõe ao paciente adquirir conhecimento para realizar o autocuidado em domicílio	0	0	2	3	10	86.66
D - O manual é efetivo quando propõe ao paciente adquirir informações sobre o processo de tratamento (etapas da radioterapia)	0	0	2	3	10	86.66
E - Aborda os assuntos mais pertinentes para o paciente com câncer de cabeça e pescoço submetido à radioterapia	0	0	0	4	11	100.00
F - Está adequado para ser utilizado como forma de tecnologia educacional na prática de profissionais da saúde	0	0	1	3	11	93.33
Total	0	0	5	17	68	94.44

*Inappropriate

†Partially appropriate

‡I am not sure

§Appropriate

||Totally appropriate

¶Agreement rate calculated by adding up the number of appropriate and totally appropriate expert judgments: TA+A x 100/total answers

## Discussion

Concerning the validation process, the professional diversity of the experts was very favorable, as it grouped different forms of expert knowledge on the theme the material addressed, resulting in multidisciplinary and comprehensive work, as observed in a study that validated an educative game for dietary orientations to diabetes mellitus patients^10^. In addition, the multidisciplinary approach is fundamental in the treatment of these patients, in view of the complexity of the therapeutic modalities and possible acute and/or late complications^17^.

In general, the experts' opinions agreed, as can be observed in the tables. The items in the first block (Table 1) refer to the intended goals, targets or ends of using the material. After the data analysis, it could be verified that the educative manual was considered valid regarding its capacity to achieve the intended purpose. Educative technologies can be effective as health education strategies during nursing care, offering possibilities to facilitate the orientations provided to the patients, and even to standardize the orientations to be provided to a certain population^8^ ^,^ ^18^.

To participate in the study, experts from the CACON/HUB and other institutions were selected to analyze whether the educative manual attends to the objectives of radiation therapy services and, hence, if its use could be extended to other institutions. In the first assessment block, item G reached a borderline agreement rate (80%). The three experts who marked option PA and N justified their choice in view of the particularities that exist at the services and in the populations from different regions of the country. Although the manual was considered accessible and understandable to different sociocultural groups, each health institution has its particularities, deriving from the treatment and the related care. Therefore, the manual can be adapted and used by nurses at other institutions whose treatment and/or population differ from the CACON/HUB.

The choice of the theme for the educative manual derived from reflections on cancer in the head and neck region and from the huge impact it entails in the patients' daily life, due to the changes the disease itself causes as well as the toxicity of the therapeutics^2^. In that context, the nursing consultation is fundamental to supply orientations on the treatment and the self-care measures needed to prevent or minimize its adverse effects. The educative manuals are important tools to guide and systemize these actions^19^. 

The second assessment block (Table 2) shows the experts' judgment on the presentation strategy of the information in the manual, including the general organization, coherence and format. The opinion of the participants graduated in languages and advertising was fundamental, as they assessed the linguistic and didactic aspects and appearance of the material in the educative manual.

Item B reached an AR of 73.33%, below the target set, in view of the underprivileged sociocultural level of the patients attended at the CACON/HUB. The concern is related to illiterate patients. Thus, the participants' considerations were accepted and considered the replacement of terms to make the reading clearer, simpler and more objective, making the language in the manual more appropriate. Examples of these replacements are the more simplified explanation on the locations the head and neck cancer affected, choosing to use the terms "nasal cavity", "pharynx", "larynx" and "oral cavity", besides including a figure to illustrate these locations. In the topic on the adverse effects of the radiation therapy, the term "xerostomia" was replaced by "dry mouth". In addition, the recommendation regarding the association of texts and illustrations was also accepted, including legends with the pictures and indicating the figures along the text contents.

Using language accessible to all social layers is fundamental, independently of the target population's instruction level, considering that the material needs to be easy to understand^18^. That is one of the reasons for using illustrations throughout the material. The AR for the item related to the appropriateness of the illustrations reached 93.33%. The use of images is important because it transforms the textual information into visual language, as a way to stimulate the interest in reading and to facilitate its understanding^18^. Colored and happy illustrations were used, in the attempt to guarantee less impressive, more relaxed and animated material. Images play an important role in communication. Therefore, the pictures used were taken from the treatment environment of the patients submitted to the radiation therapy, representing this population's actual scenario. 

The increasing use of educative materials as resources in the health education process created new possibilities for interaction among nurses, patients and families^20^. Nevertheless, it is highlighted that the educative manual does not replace the nurses' verbal orientations during the nursing consultation, although it would be very useful to strengthen the recommendations transmitted. Thus, this tool can be useful for in-home consultations after nurses have provided their orientations. 

In that context, the selected information for inclusion in the manual should be truly fundamental for the material to be significant, attractive, concise and objective. Explanations on possible adverse reactions of the treatment can enhance the patients' understanding and satisfaction without inducing increased anxiety^21^. Therefore, the intention was to encourage the reading through the use of friendly and patient-oriented language, also as a way to include it in the health/disease process. 

The end result of the elaboration and improvement of the manual therefore contained essential information for patients with head and neck cancer undergoing radiation therapy, as well as illustrations coherent with the text, favoring nursing communication and the understanding of whoever uses the material. 

Some suggestions were not fully accepted in the educative material after analyzing scientific evidences. One example was the aspect on oral hygiene, in which one of the experts suggested summarizing the topic in the educative material on the theme, while another questioned the need to present teeth brushing in detail. In a systematic review by the oral mucositis study of the Multinational Association of Supportive Care in Cancer (MASCC), da International Society of Oral Oncology (ISOO), oral hygiene was considered the most evidenced protocol to prevent oral mucositis in different treatment modalities, including radiation therapy, chemotherapy or hematopoietic stem cell transplantation^22^. 

Therefore, the decision was made to maintain the detailed description and illustration on how oral hygiene should be executed (the ideal times and tools needed for brushing, as well as the step-by-step for the correct execution of this action), considering that oral hygiene protocols present the highest evidence levels to prevent oral mucositis in cancer patients of any age range^22^ ^-^ ^24^. 

Also regarding care for the oral cavity, the decision was made to remove the topic that indicated rinsing with 0.12% chlorhexidine digluconate, due to the lack of scientific evidence to establish this protocol. Although studies evidence the reduction of mucositis and ulceration in chemotherapy patients, no significant results are presented for patients submitted to radiation therapy^22^ ^-^ ^24^. Therefore, a conflict exists in the literature on the use of chlorhexidine, and the MASCC/ISOO does not recommend its use to prevent and/or treat oral mucositis in head and neck cancer patients undergoing radiation therapy^22^. In view of this panorama, it cannot be defined yet what therapeutic modality is the most indicated as a protocol to prevent and/or treat oral mucositis. Therefore, the decision was made to remove the indication of rinsing from the educative manual, leaving this measure up to each institution.

The third block (Table 3) on the characteristics that turn the manual into relevant material, also reached the minimum agreement rate determined. This fact confirms the importance of using the educative material to contribute to the promotion of health education for head and neck cancer patients submitted to radiation therapy, and to strengthen the orientations provided during nursing consultations.

Offering information and orientations to patients and their relatives through printed educative material can be an important strategy to enhance the treatment compliance and to facilitate the patients' acquisition of coping and decision making skills^25^. When the patients take home material with the orientations given during the nursing consultation, the transmission of this information can continue beyond the hospital context, disseminating them at home among the patients' caregivers and relatives. In addition, the education can be ongoing, as the patients have material they can consult whenever they are in doubt or feel anxious.

As the 100.00% agreement rate of items A, B and E in Table 3 illustrates, the educative manual was considered pertinent with regard to its approach of the needs and demands of head and neck cancer patients undergoing radiation therapy, and also regarding the information and orientations the tool incorporates.

This study is limited by the lack of validation by the target population. Therefore, further study has been planned to proceed with the validation of the educative material through the semantic assessment of the material by head and neck cancer patients submitted to radiation therapy.

The material can help the patients to understand the therapeutic process they are submitted to during the radiation therapy, thus contributing to self-care. In addition, it can be used as a teaching strategy to support clinical practice during nursing consultations. Studies are needed to assess the efficacy of the manual to produce behavioral changes in terms of self-care compliance. 

## Conclusion

The face and content validation of the educative manual was achieved, which was considered relevant for head and neck cancer patients submitted to radiation therapy. The construction of the educative manual was based on scientific knowledge, available in the current literature, as well as on the participants' suggestions, who contributed to the final version of this material.
